# Survivomics: natural product clues from virus-unaffected crops for next-generation plant immunity

**DOI:** 10.3389/fpls.2026.1796880

**Published:** 2026-04-10

**Authors:** Pradeep Pant

**Affiliations:** Department of Biotechnology, Bennett University, Uttar Pradesh, India

**Keywords:** antivirals, crop protection, plant immunity, plant viruses, survivomics

## Introduction

In an era where one in every ten people goes to bed hungry, and global food demand is expected to rise by 60% by 2050, we are challenged by food scarcity alongside food vulnerability (Iriti and Vitalini, 2020). While biotechnology, genome editing, and synthetic agrochemicals have advanced food production, over $220 billion worth of crops are lost each year due to plant pathogens, particularly viruses (Hinge et al., 2021; Devi et al., 2022; Faizal et al., 2024). These are not simply biological irritants but molecular saboteurs, exacting heavy economic and nutritional tolls on agriculture-dependent communities.

But amid this devastation lies an ecological mystery. Why does one crop fall while another stands? Walk through a virus-affected farm: tomato vines wilt and spot, zucchinis curl into grotesque forms, while potato plants stand green and unbothered. A virus like Zucchini yellow mosaic virus (ZYMV) ravages its host, but the tomatoes or beans in the adjacent rows remain pristine. It has been observed that plant viruses display diverse natural host ranges: while some are restricted to specific plant species or genera (cacao swollen shoot virus, citrus tristeza virus, and rice tungro bacilliform virus), others such as cucumber mosaic virus and tobacco mosaic virus are capable of infecting a wide range of hosts across multiple plant families (Descriptions of plant viruses; Dawson et al., 2013; Friscina et al., 2017; Kimaru et al., 2020). This diversity suggests that compatibility between viral infection strategies and host cellular environments plays a key role in determining susceptibility. We believe that this specificity or range selectivity is not merely botanical luck; it’s biochemical design. What if the uninfected crops are not just “not targeted”, but actively resisting? What if their phytochemical or biomolecular environment is hostile to the virus through inhibitory secondary metabolites, RNA-based immune signals, or structural defenses in the plant cell walls?

We refer to this concept as “Survivomics,” defined here as the systematic exploration of biomolecular and metabolic traits present in plants that remain unaffected under pathogen pressure while neighboring susceptible crops become infected. By focusing on the biochemical environment of these naturally resilient species, Survivomics aims to identify metabolites or molecular mechanisms that may contribute to antiviral resistance. This hypothesis, though nascent, calls for serious exploration. Many plants untouched by viruses may harbor natural antivirals, compounds that interfere with viral coat proteins, inhibit RNA replication, or modulate host-virus interactions. Yet these biochemical fortresses are poorly mapped, their contents uncharacterized.

## A reverse-engineering approach from nature’s inventory

**Figure 1:**
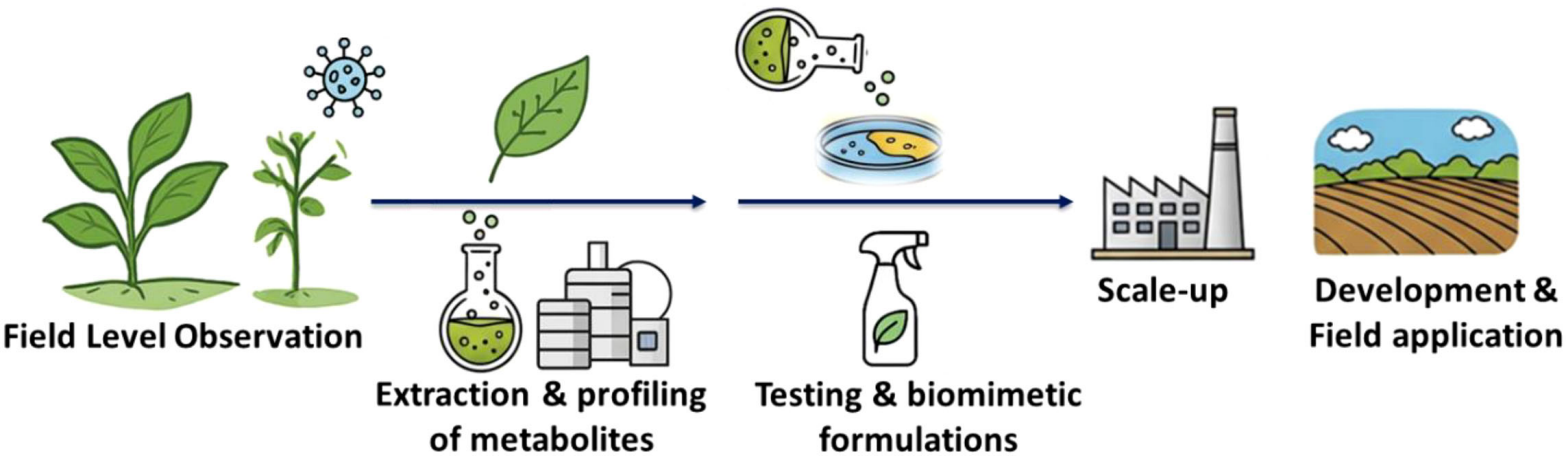
Workflow illustrating the stepwise approach for identifying natural antiviral agents in plants, starting from field-level observations to biomimetic formulation development.

We propose a reversal in how plant antivirals are discovered. Instead of starting with viral targets and designing inhibitors synthetically, we start with what survives, using comparative metabolomics and transcriptomics to identify protective biomolecules. While synthetic molecules that are novel to nature may show initial effectiveness, their accumulation poses hazardous risks, and their management has become a daunting challenge (Naidu et al., 2021). Several such synthetic molecules along with natural compounds, such as abscisic acid, oligosaccharins, ningnanmycin, S-methylbenzo[1,2,3]thiadiazole-7-carbothioate, dufulin, flavonoids, cytosinpeptidemycin, tiadinil, Gu188, 3-acetonyl-3-hydroxyoxindole (AHO), methiadinil, vanisulfane, antofine, and chloroinconazide, have already been explored (Havsteen, 2002; Song et al., 2010; Hemberg, 1949; Callow, 1993; Xiang et al., 1995; Kunz et al., 1997; Wu et al., 2003; Michiko et al., 2004; Lv et al., 2007; Li et al., 2008; Fan et al., 2009; Han et al., 2014; Xin et al., 2019; Lv et al., 2020; Meng et al., 2020; Mo et al., 2024). The proposed pipeline addresses this issue by harnessing compounds from nature’s own inventory. The proposed pipeline for discovering and applying natural plant-derived antiviral agents is shown in **Figure 1**. It begins with field-level observations, where unaffected crops growing alongside infected ones under identical conditions are identified as potential sources of resistance. These plants undergo high-throughput metabolite profiling using advanced techniques such as LC-MS and NMR to detect unique bioactive compounds. The identified metabolites are then tested through functional assays to evaluate their impact on viral replication and plant infection models. Finally, promising candidates are translated into biomimetic formulations, such as sprays or seed coatings, designed to replicate natural defense mechanisms and provide practical field-level protection against plant viruses.

Several plant-derived secondary metabolites have been reported to interfere with plant virus infections. For example, plant derived compounds, such as ursolic acid (UA) and 4-methoxycoumarin, have demonstrated antiviral properties against tobacco mosaic virus, wherein their antiviral effects were comparable to the commercial agent lentinan (Cai et al., 2020). Flavonoids, such as naringin quercetin and vitexin, have been shown to reduce viral accumulation in infected plants (Krcatović et al., 2008; Li et al., 2025). What remains is their contextual, crop-specific, and virus-targeted exploration in a systems biology framework. The central dogma of agricultural biotechnology has been innovation: create something new. Yet evolution is the greatest innovator. It has already conducted billions of years of molecular trials, and nature’s survivors are already carrying antidotes encoded not in patents, but in their metabolomes.

This Perspective invites the plant natural products community to pivot: from exclusive innovation to evolution-guided inspiration. The next generation of antiviral agrochemicals might not be designed, they might be discovered, hidden in the roots, leaves, and fruits of the virus-resistant species we too often overlook.

## Discussion

Despite modern advances in agriculture, over 40% of global crop production is lost annually due to pests and plant diseases, where viruses being silent yet devastating agents in this biological warfare. Paradoxically, in the same field, where a virus can decimate one crop, others may thrive unscathed. This Perspective explores the underappreciated phenomenon of virus-specificity in plants: why some crops fall while others flourish. We argue that the answer may lie in the unique phytochemical and biomolecular arsenals of unaffected plants. Nature, we suggest, has already developed virus-resistance blueprints, encoded in the secondary metabolomes of resilient species.

It is important to recognize that the absence of infection in certain crops may arise from multiple factors beyond intrinsic biochemical resistance. Differences in virus transmission routes, including vector specificity (e.g., aphids, whiteflies, thrips, nematodes), mechanical transmission, or environmental constraints, can strongly influence which plants become infected in a given field. Therefore, the Survivomics framework should be viewed as a complementary strategy aimed at identifying potential biochemical contributors to resistance while acknowledging that ecological and epidemiological factors also shape virus distribution. Further, plant antiviral defense is multifaceted and includes several well-characterized mechanisms beyond secondary metabolism. These include pathogenesis-related (PR) proteins, systemic acquired resistance (SAR), RNA interference mediated by small interfering RNAs, and virus cross-protection mechanisms in which mild viral strains can limit infection by more severe variants. The Survivomics framework does not replace these established defense paradigms but instead complements them by focusing on the potential role of plant metabolomes in shaping virus–host compatibility.

Rather than racing to reinvent synthetic molecules, we advocate for a paradigm shift toward systematic mining of natural product profiles from virus resilient crops to identify broad spectrum plant antivirals or bioprotective agents. Exploring these naturally occurring defense strategies offers a path toward sustainable crop protection with reduced ecological burden.

While the Survivomics framework provides a promising strategy for identifying naturally occurring antiviral compounds, it may not be universally applicable across all crop–virus systems. Variations in plant physiology, virus host range, and environmental conditions may influence the feasibility of identifying transferable biomolecular defenses. Therefore, this approach should be viewed as a complementary strategy that can work alongside existing genetic, agronomic, and biotechnological interventions for crop protection.

Food security depends as much on minimizing losses as on increasing production. As plant viruses continue to compromise yields worldwide, research attention must extend beyond vulnerable crops to those that persist under identical environmental and pathogenic pressures. Understanding and translating these survival strategies into practical interventions may provide durable solutions for future agricultural resilience.
